# Identification and Regulatory Roles of a New Csr Small RNA from Arctic Pseudoalteromonas fuliginea BSW20308 in Temperature Responses

**DOI:** 10.1128/spectrum.04094-22

**Published:** 2023-01-10

**Authors:** Jiao Wen, Li Liao, Zedong Duan, Shiyuan Su, Jin Zhang, Bo Chen

**Affiliations:** a Key Laboratory for Polar Science, Ministry of Natural Resources, Polar Research Institute of China, Shanghai, China; b School of Oceanography, Shanghai Jiao Tong University, Shanghai, China; c Southern Laboratory of Ocean Science and Engineering (Guangdong, Zhuhai), Zhuhai, China; Connecticut Agricultural Experiment Station

**Keywords:** *Pseudoalteromonas*, sRNA, CsrA, temperature, regulation

## Abstract

Small RNAs (sRNAs) play a very important role in gene regulation at the posttranscriptional level. However, sRNAs from nonmodel microorganisms, extremophiles in particular, have been rarely explored. We discovered a putative sRNA, termed Pf1 sRNA, in Pseudoalteromonas fuliginea BSW20308 isolated from the polar regions in our previous work. In this study, we identified the sRNA and investigated its regulatory role in gene expression under different temperatures. Pf1 sRNA was confirmed to be a new member of the CsrB family but has little sequence similarity with Escherichia coli CsrB. However, Pf1 sRNA was able to bind to CsrA from E. coli and *P. fuliginea* BSW20308 to regulate glycogen synthesis. The Pf1 sRNA knockout strain (Δ*Pf1*) affected motility, fitness, and global gene expression in transcriptomes and proteomes at 4°C and 32°C. Genes related to carbon metabolism, amino acid metabolism, salinity tolerance, antibiotic resistance, oxidative stress, motility, chemotaxis, biofilm, and secretion systems were differentially expressed in the wild-type strain and the Δ*Pf1* mutant. Our study suggested that Pf1 sRNA might play an important role in response to environmental changes by regulating global gene expression. Specific targets of the Pf1 sRNA-CsrA system were tentatively proposed, such as genes involved in the type VI secretion system, TonB-dependent receptors, and response regulators, but most of them have an unknown function. Since this is the first study of CsrB family sRNA in *Pseudoalteromonas* and microbes from the polar regions, it provides a novel insight at the posttranscriptional level into the responses and adaptation to temperature changes in bacteria from extreme environments. This study also sheds light on the evolution of sRNA in extreme environments and expands the bacterial sRNA database.

**IMPORTANCE** Previous research on microbial temperature adaptation has focused primarily on functional genes, with little attention paid to posttranscriptional regulation. Small RNAs, the major posttranscriptional modulators of gene expression, are greatly underexplored, especially in nonpathogenic and nonmodel microorganisms. In this study, we verified the first Csr sRNA, named Pf1 sRNA, from *Pseudoalteromonas*, a model genus for studying cold adaptation. We revealed that Pf1 sRNA played an important role in global regulation and was indispensable in improving fitness. This study provided us a comprehensive view of sRNAs from *Pseudoalteromonas* and expanded our understanding of bacterial sRNAs from extreme environments.

## INTRODUCTION

The majority of the biosphere on Earth is permanently below 5°C, primarily in the polar regions and the deep ocean. The cold environments are dominated by microorganisms which form the base of the food web (1). Microorganisms living in the cold biosphere have evolved sophisticated strategies to adapt to low temperatures (2). Meanwhile, climate change and global warming have become a threat to the biosphere, particularly the cold biosphere of the polar regions. The polar regions are most vulnerable to climate change and thus become hot spots of global warming (3). Therefore, understanding the responses and adaptation potential of cold-adapted microorganisms to rising temperatures is critical. Many studies have been conducted to investigate the adaptation of polar microorganisms to extreme environments and their changes at the genomic, transcriptomic, and proteomic levels (4–6). However, little is known about the role of posttranscriptional regulation and the functions of small RNAs (sRNAs) in adaptation to extreme environments and climate change in polar microorganisms.

sRNAs are emerging as important posttranscriptional regulators of gene expression (7–10). In general, sRNAs refer to noncoding regulatory RNAs, usually with 50 to 500 nucleotides. Once thought to be nonfunctional transcription noise, sRNAs have now been found in bacteria, archaea, and eukaryotes (11). Compared with protein-mediated transcriptional regulation, sRNA-mediated gene regulation is energy efficient, responds to changes more quickly and sensitively, adapts to evolutionary needs faster, and can achieve one to many regulations (12, 13). As a result, posttranscriptional regulation mediated by regulatory sRNAs has become a key player in fine-tuning gene expression to environmental stimuli. Most sRNAs function as base-pairing regulators (14). However, Csr sRNAs function as an antagonist of CsrA, a global regulatory protein discovered for its effects on central carbon metabolism (15, 16). The Csr (carbon storage regulator) system, comprised of Csr sRNA and CsrA, has been found to play a global regulatory role in a variety of complex physiological processes, including carbon metabolism (17), amino acid metabolism (18), osmotic pressure regulation (19), motility (20), chemotaxis (21), virulence (22), biofilm formation (23), quorum sensing (24), stress responses (16), and CRISPR-Cas immunity (25). Csr sRNAs have been studied primarily in E. coli (9, 16) and pathogens such as Salmonella enterica (26), Pseudomonas fluorescens (27), Pseudomonas aeruginosa (28), and Vibrio cholerae (29). However, much less is known about Csr sRNAs in nonmodel nonpathogenic microorganisms, which are the unknown majority in the environment. In particular, the discovery of Csr sRNAs and their regulatory roles in cold-adapted bacteria from the polar regions have received little attention.

*Pseudoalteromonas*, which has been used as a cold adaptation model (30), is an excellent choice for studying the diversity of sRNA and its global regulatory roles in polar microorganisms. *Pseudoalteromonas* is a dominant bacterial group in the polar regions, well adapted to cold temperatures, and a key species in ocean carbon export and interactions with higher organisms (31–33). We previously performed whole-genome sequencing and transcriptomics on Pseudoalteromonas fuliginea BSW20308, a pyschrotolerant bacterium that is common in seawater and that dominates in polar oceans (34, 35). Surprisingly, we discovered that expression of transfer-messenger RNA, 6S RNA, and an unknown intergenic sequence increased dramatically at 32°C compared to 4°C (35). We hypothesized that this unknown intergenic sequence contained a putative unknown sRNA (tentatively dubbed Pf1 sRNA) and that Pf1 may play an important regulatory role in temperature stress responses. To confirm and identify this putative Pf1 sRNA, we conducted experiments to ascertain what Pf1 is, whether it is functional, and what roles Pf1 plays in temperature responses. This study provides comprehensive insight into the newly discovered sRNA from *Pseudoalteromonas* in the polar regions and sheds light on its posttranscriptional regulation in response to environmental changes.

## RESULTS

### Identification and sequence analysis of Pf1 sRNA.

**FIG 1:**
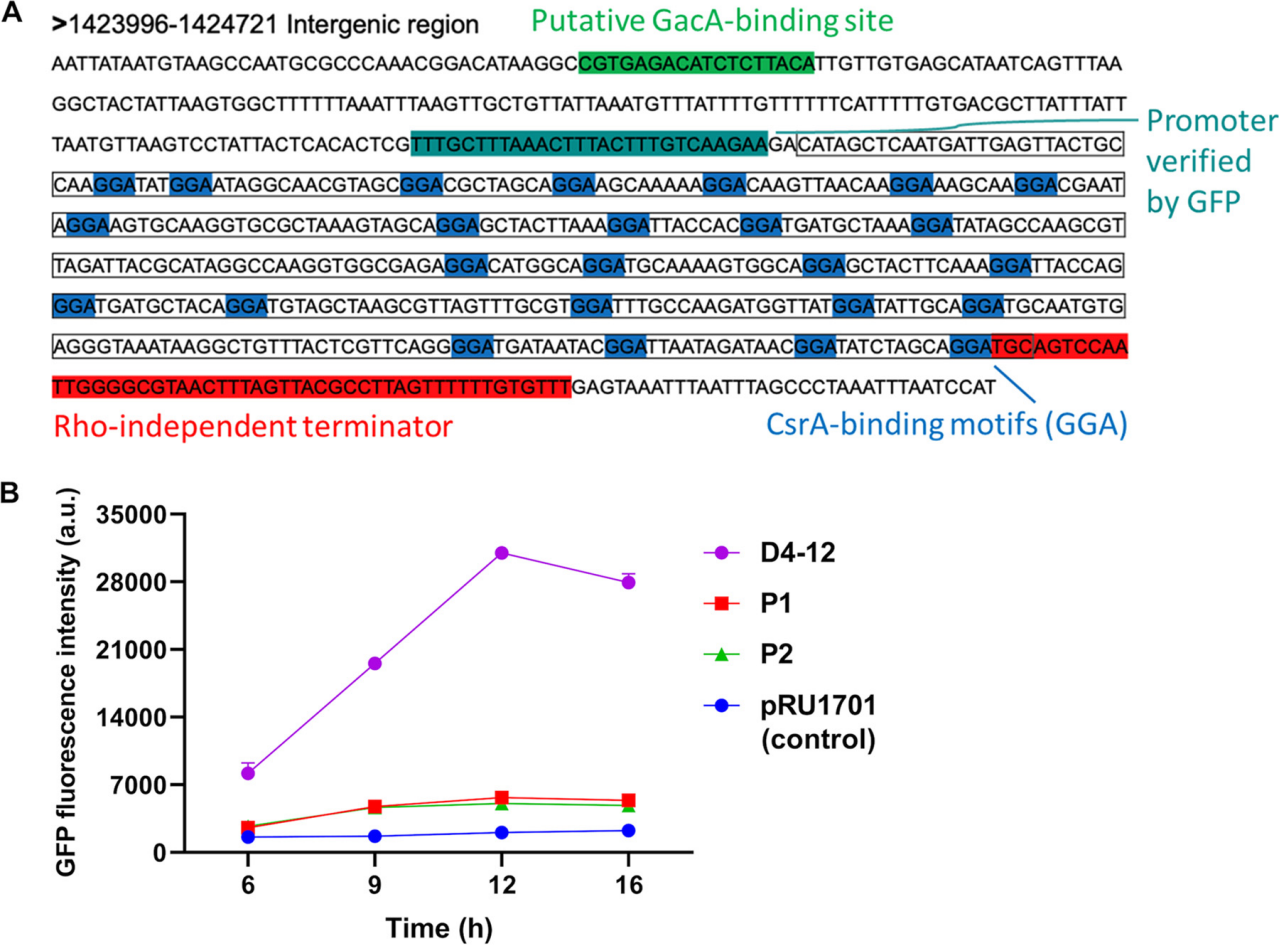
Identification of Pf1. (A) Pf1 sequence and its characteristics; (B) predicted promoter activity validation and minimum promoter sequence identification by fluorescence measurement. The promoter sequences tested are listed in Data Set S1 in the supplemental material, and the corresponding GFP fluorescence intensities are in Fig. S1.

**FIG 2:**
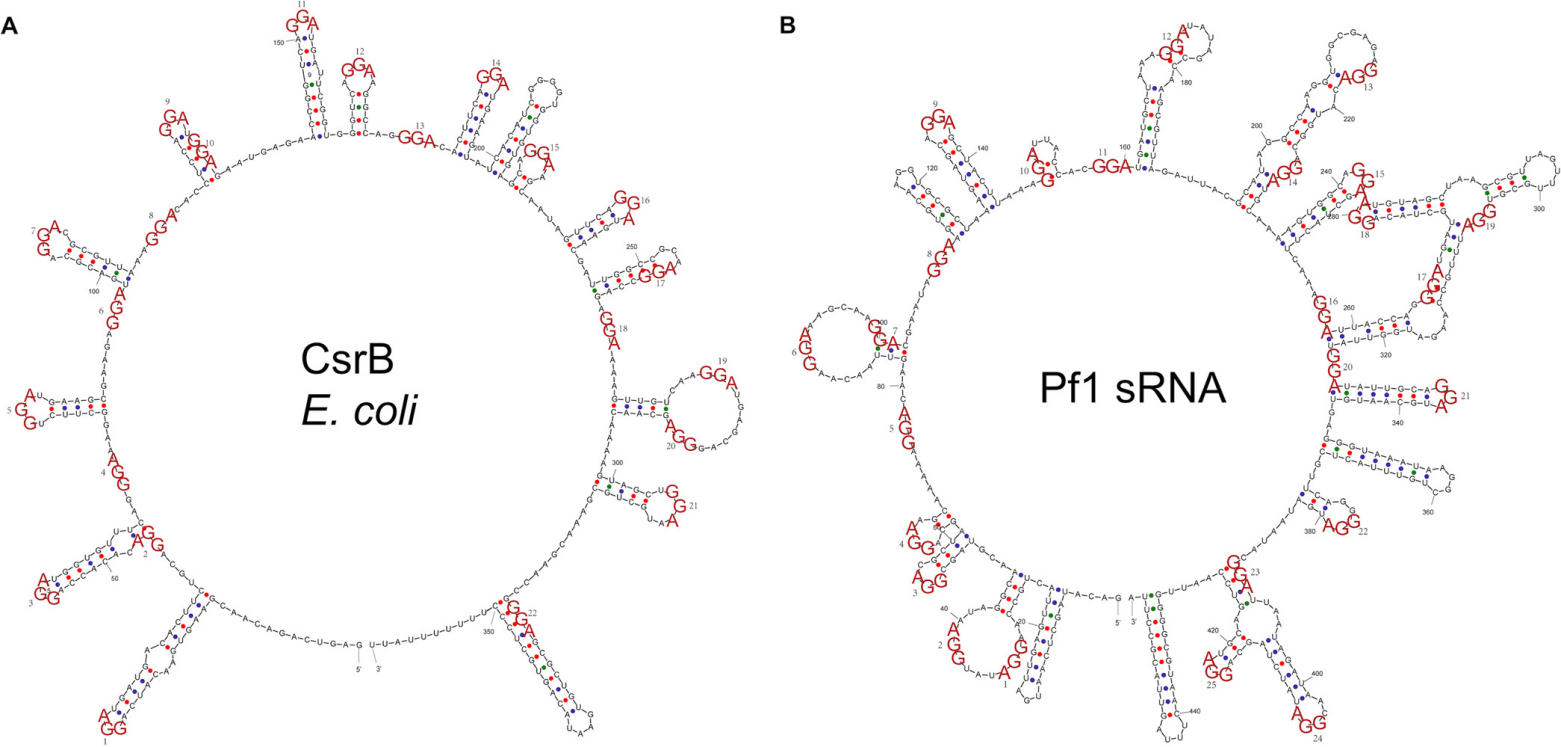
Secondary structure comparison of E. coli CsrB (A) and Pf1 (B).

**FIG 3:**
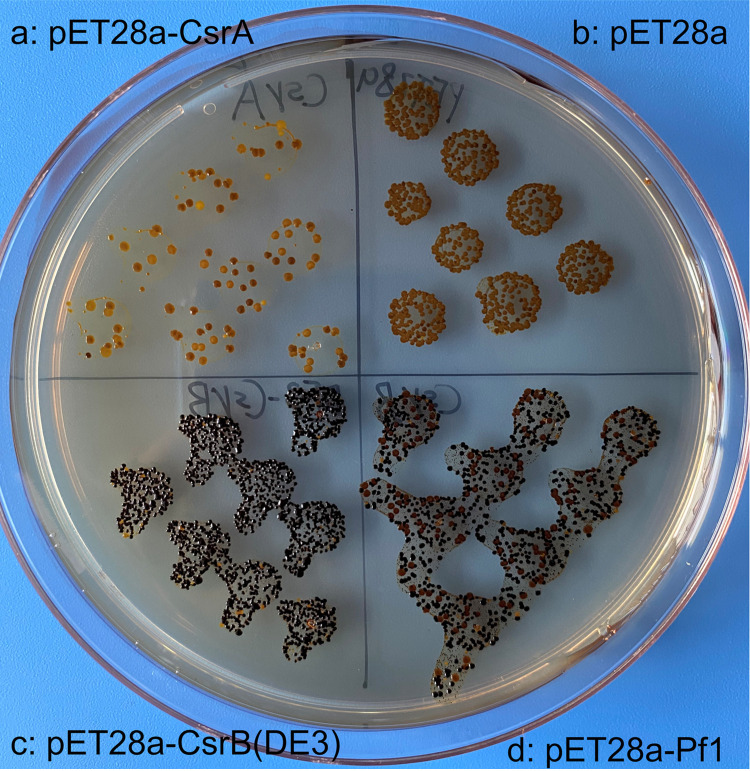
Interaction of Pf1 with CsrA in E. coli DE3 and its role in glycogen synthesis evaluated by iodine staining. The more glycogen produced, the darker the colony stained by iodine. (a) E. coli DE3 containing pET28a cloned with CsrA from BSW20308; (b) E. coli DE3 containing pET28a; (c) E. coli DE3 containing pET28a cloned with CsrB from itself; (d) E. coli DE3 containing pET28a cloned with Pf1.

Promoters and a rho-independent terminator were predicted in the Pf1 sRNA putative coding region with high confidence scores (Fig. 1A; see Data Set S1 in the supplemental material). All sequences extracted from the predicted promoters were able to initiate the expression of a promoter-less green fluorescent protein (GFP) gene, with the shortest sequence, D4-12, being the strongest promoter (Fig. 1B; Fig. S1). A putative GacA-binding sequence (CGTGAGACATCTCTTACA [underlining indicates that the base is conserved]) and 21 putative CsrA-binding motifs (GGA) were found in the hypothesized Pf1 sRNA coding region (Fig. 1A). Moreover, a high probability (0.96) of being an sRNA of the CsrB/C family was given by the algorithm InvenireSRNA. According to the predicted secondary structure of Pf1, there were multiple loops formed by the sequences AGGA/UGGA/AGGGA, with the exception of the terminator, which is similar to that of E. coli CsrB (Fig. 2) (36). However, the secondary structure of Pf1 sRNA (Fig. 2B) is more complex, with secondary or tertiary loops or larger loops, than that of E. coli CsrB (Fig. 2A). Together, the data suggest that Pf1 sRNA is most likely a new sRNA of the CsrB family. To further confirm the prediction, Pf1 sRNA was cloned into pET28a and transformed into E. coli DE3 to test its binding to CsrA by following previous protocols (37). Overexpression of CsrA from strain BSW20308 inhibited glycogen synthesis, as evidenced by the lightest color developed after iodine staining (Fig. 3). As a result, it confirmed that Pf1 bound to CsrA in E. coli DE3 and regulated glycogen synthesis, as is typical of Csr sRNAs. Therefore, Pf1 was identified as a new Csr sRNA.

To understand the origin and distribution of Pf1-like sRNAs, the Pf1 sRNA sequence was then compared to the NCBI NR database, yielding 260 hits, with similarities ranging from 73.53% to 100%. Interestingly, all 260 hits were from *Pseudoalteromonas* and were most likely Csr sRNAs due to the high sequence identity and the presence of dense GGA motifs. In general, Pf1-like sequences were more similar within species than across species and tended to cluster according to species on the phylogenetic tree (Fig. S2). However, there were outliers, with sequences from distinct species intertwined (Fig. S2). The majority of hits were clustered in one clade on the 16S rRNA gene tree (Fig. S3). A few hits were scattered across other branches (Fig. S3). No significant sequence identity was found between Pf1 sRNA and any other well-studied Csr sRNAs from species like E. coli. Therefore, Csr sRNAs varied greatly in sequence across genera, hindering their discovery.

### Comparative transcriptomics and proteomics.

**FIG 4:**
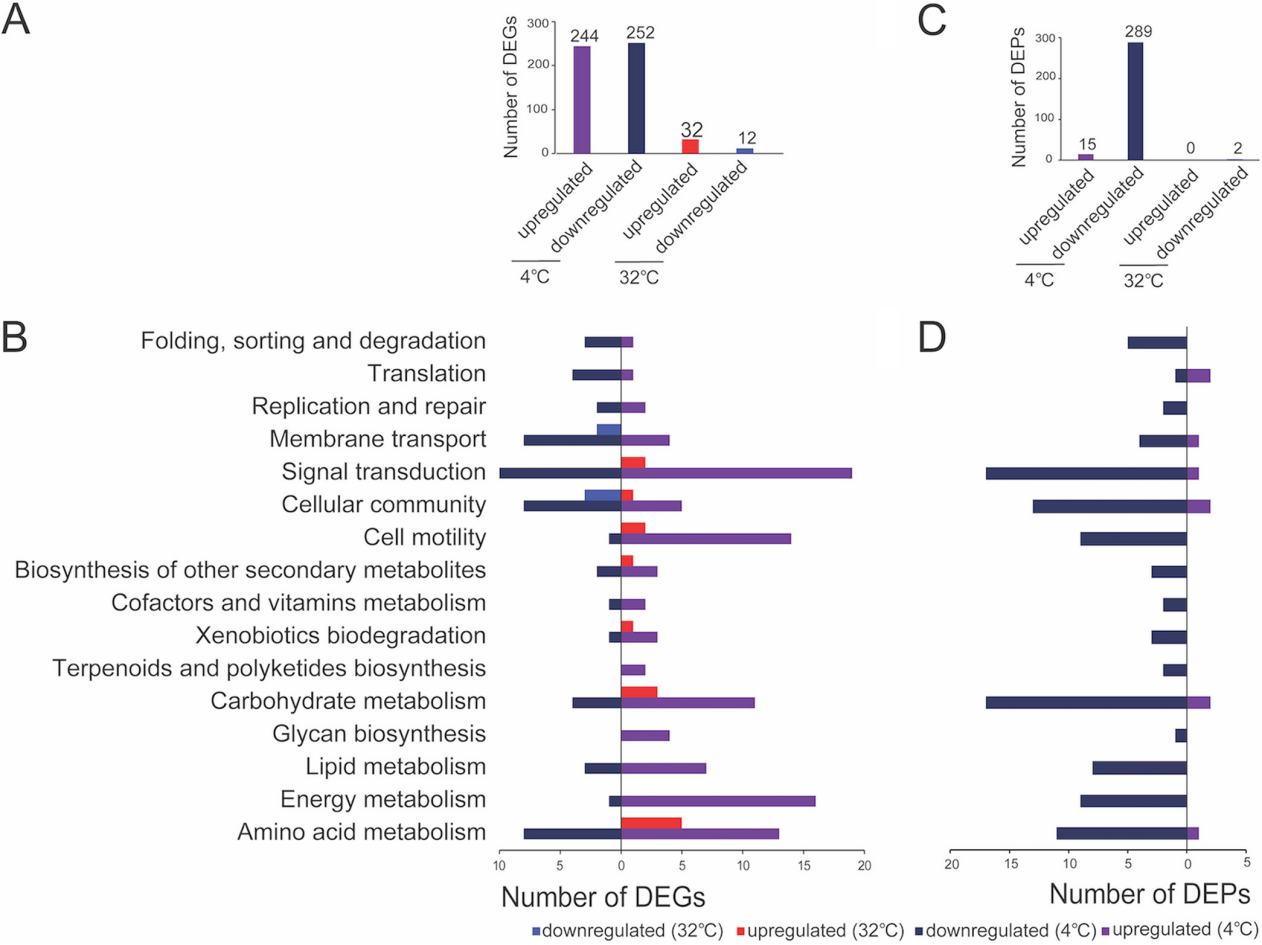
Comparative transcriptomics and proteomes of the Δ*Pf1* mutant normalized to those of the WT at 4°C and 32°C. (A) Number of DEGs at 4°C and 32°C; (B) KEGG pathway categories of DEGs at 4°C and 32°C; (C) number of DEPs at 4°C and 32°C; (D) KEGG pathway categories of DEPs at 4°C and 32°C.

To better understand the functions of Pf1 sRNA in response to temperature changes, a knockout mutant (Δ*Pf1*) was constructed. Transcriptomics and proteomics were performed on biological triplicates of the wild-type strain (WT) and the Δ*Pf1* mutant grown to stationary phase at 4°C and 32°C. The WT strain was used as a control to identify differentially expressed genes (hereafter referred to as DEGs) (Data Set S2A to D) and differentially expressed proteins (hereafter referred to as DEPs) (Data Set S3A to D) in the Δ*Pf1* mutant. Similar to previous studies (38, 39), the overlap between the transcriptomic data and the proteomic data was relatively low, containing 63 functional genes (Data Set S4). Consistent with the transcriptome data, significant DEPs were mostly detected at 4°C, with most of them being downregulated (Fig. 4). The dominant KEGG categories of DEGs and DEPs were similar (Fig. 4). More genes were affected at low temperature (4°C) than at high temperature (32°C) in the absence of Pf1 (Fig. 4). The overlap of DEGs and DEPs at 4°C and 32°C was low (Fig. S4), with most of the DEGs and DEPs being temperature specific. A full list of DEGs and DEPs detected specifically at 4°C and 32°C are available in Data Set S5A to D.

In general, the top DEGs downregulated specifically at 4°C were mostly hypothetic proteins, suggesting that the lack of Pf1 may have some unidentified effect. In addition to hypothetical proteins, stress-related proteins such as phage shock proteins and envelope stress response membrane proteins were also among the top downregulated DEGs at 4°C (Data Set S5A). Transposases were differentially regulated at 4°C, with three IS*3* family transposases downregulated and other IS family transposases, such as those of IS*110*, IS*91*, IS*481*, and IS*30* families, upregulated (Data Set S5A). According to KEGG categories, DEGs, specifically at 4°C, were grouped mostly into the categories of signal transduction, energy metabolism, amino acid metabolism, cell motility, and carbohydrate metabolism (Fig. S4).

At 32°C, many fewer genes showed differential expression (Fig. S4). Most DEGs were upregulated at 32°C, including genes involved in flagellar biosynthesis, stress responses, and transporters (Data Set S5B). Only three DEGs were downregulated at 32°C, including a type VI secretion protein and two hypothetical proteins. According to the KEGG categories, DEGs, specifically at 32°C, were associated with amino acid metabolism, carbohydrate metabolism, secondary metabolite metabolism, cell motility, and cellular community, as well as signal transduction and membrane transport (Fig. S4).

**FIG 5:**
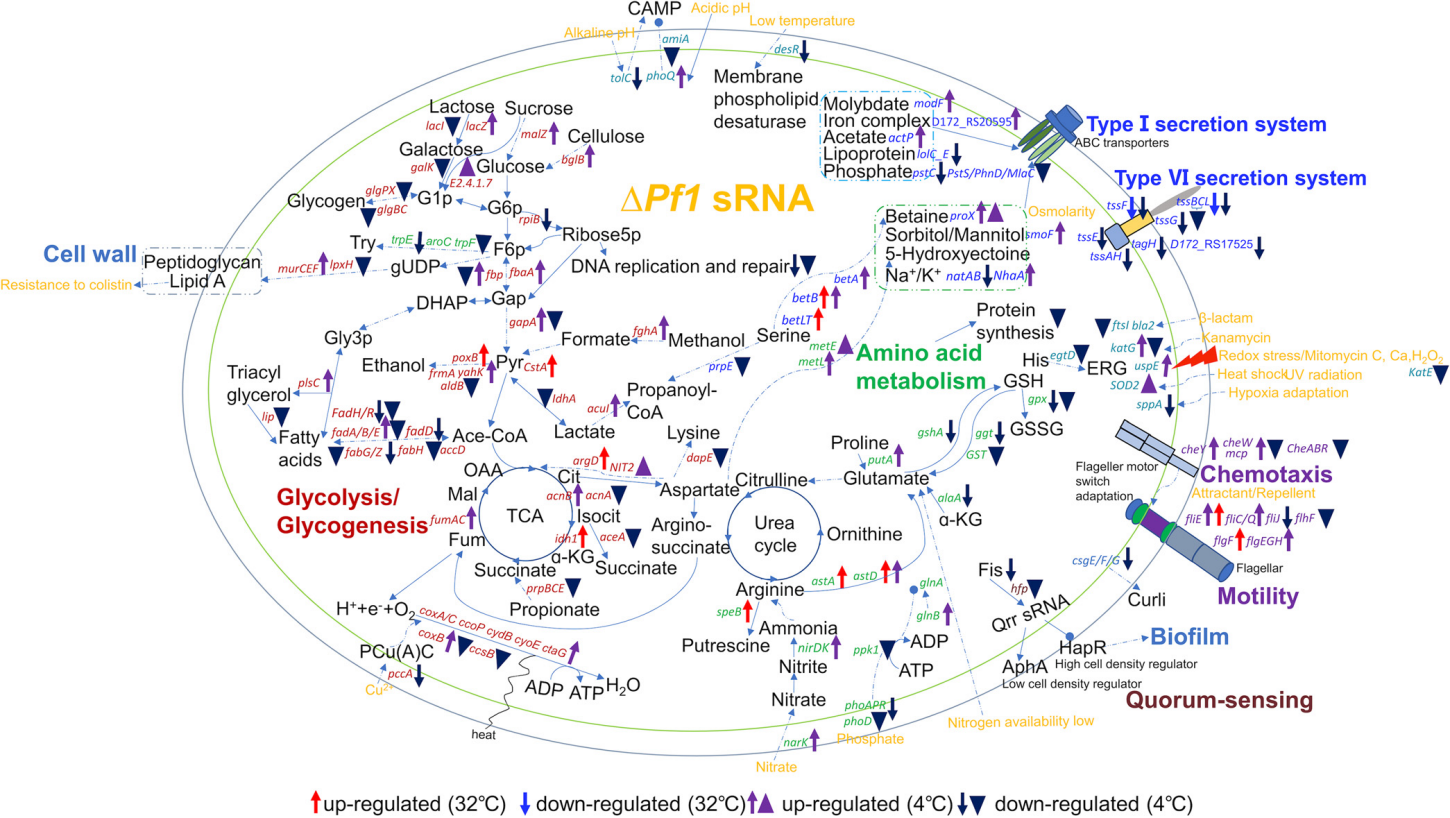
Overview of transcriptome and proteome data analysis involving upregulated and downregulated genes and proteins with known function in the Δ*Pf1* mutant. Arrows and triangles next to the genes and proteins represent the upregulation and downregulation in the transcriptome and proteome, respectively. The colors represent different modulation outcomes: red, upregulated at 32°C; blue, downregulated at 32°C; purple, upregulated at 4°C; navy blue, downregulated at 4°C. G1/6p, glucose-1/6p; F6p, fructose-6p; gUDP, UDP-GlcNAc; Try, tryptophan; Gap, glyceraldehyde-3-phosphate; DHAP, dihydroxyacetone phosphate; Gly-3p, glycerol-3p; Pyr, pyruvate; Ace-CoA, acetyl coenzyme A; Cit, citrate; Isocit, isocitrate; ɑ-KG, 2-ketoglutarate; Fum, fumarate; Mal, malate; OAA, oxaloacetate; CAMP, cationic antimicrobial peptide; ERG, ergothioneine.

All DEGs and DEPs with known functions are summarized in an overview illustration (Fig. 5), and the major processes were further examined in the following categories, with the results based mostly on DEGs or DEPs at 4°C by default, unless otherwise specified. Hereafter, we use gene symbols to refer to transcriptome data and protein symbols to represent proteome data.

**FIG 6:**
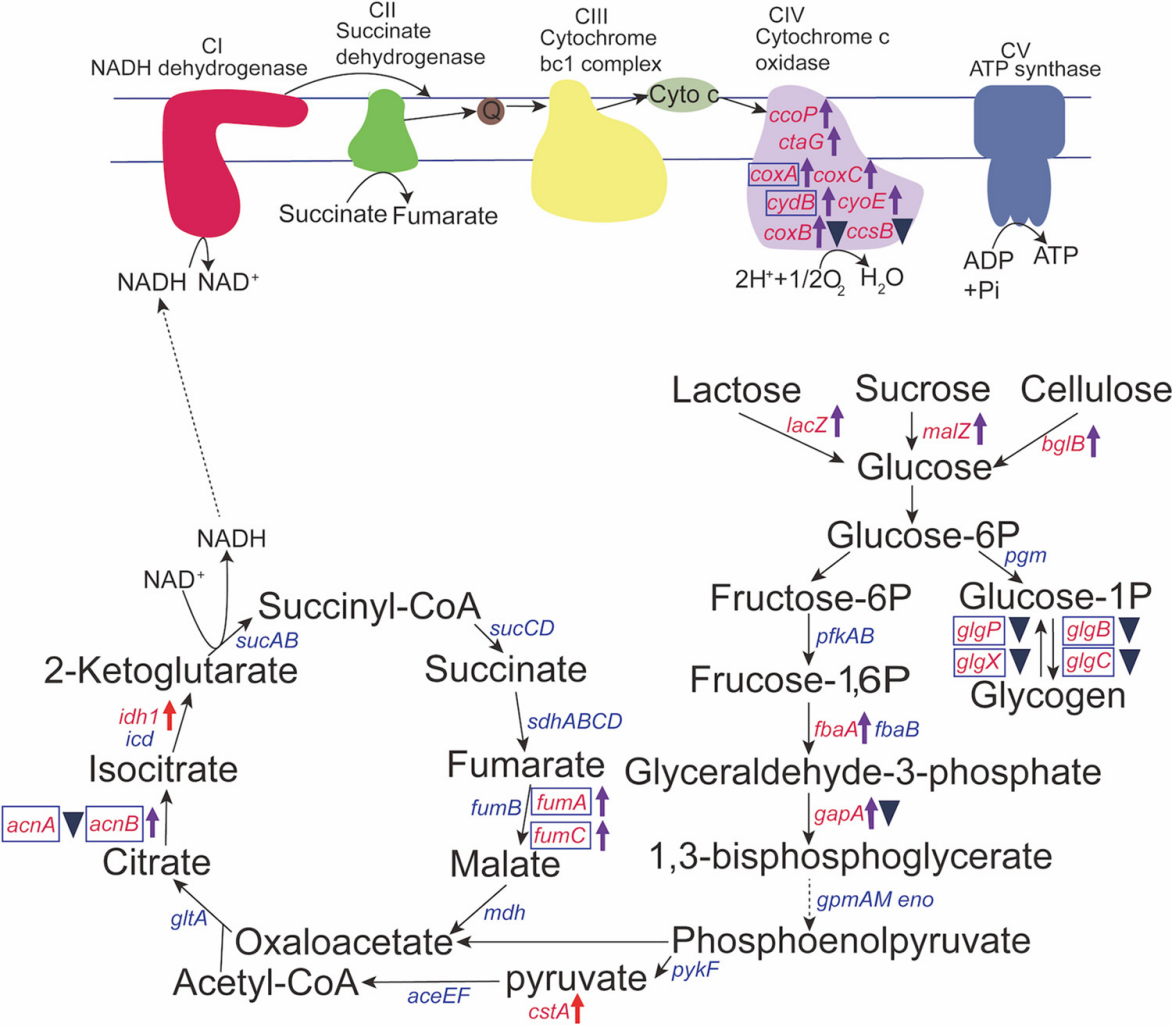
Upregulated and downregulated genes in carbon metabolism, TCA cycle, and oxidative phosphorylation in the Δ*Pf1* mutant. Genes enclosed in blue boxes represent the identified regulatory targets of CsrA in E. coli. Arrows and triangles next to the genes and proteins represent the upregulation and downregulation in the transcriptome and proteome, respectively. The colors represent different modulation outcomes: red, upregulated at 32°C; blue, downregulated at 32°C; purple, upregulated at 4°C; navy blue, downregulated at 4°C.

**(i) Carbohydrate metabolism and respiration.** Pf1 sRNA was shown to play a key role in regulating carbohydrate metabolism and respiration. In the transcriptomes, genes involved directly in glycolysis (*fbaA* and *gapA*) and genes involved in providing substrates entering glycolysis (including the lactose-degrading gene *lacZ*, the sucrose-degrading gene *malZ*, and the cellulose-degrading gene *bglB*) were all upregulated in the Δ*Pf1* mutant (Fig. 6). The highly specific pyruvate transporter *cstA*, energized by a proton gradient, was upregulated (32°C). Meanwhile, the genes *fumA*, *fumC*, *acnB*, and *idh1* (only at 32°C, a rate-limiting step) in the tricarboxylic acid (TCA) cycle were upregulated in the Δ*Pf1* mutant in the transcriptomes (Fig. 6). In addition, genes responsible for converting aspartate into the TCA cycle were upregulated, including those coding for aspartate aminotransferase (*argD*, only at 32°C) and amidohydrolase NIT2 (Fig. 5). Genes responsible for cytochrome *c* oxidase biosynthesis and assembly (*coxABC* encoding a-type subunits I, II, and III, *ccoP* encoding cbb3-type subunit III, and *ctaG* encoding the assembly protein), cytochrome *d* ubiquinol oxidase (*cydB*), and protoheme IX farnesyltransferase (*cyoE*) were all upregulated in the Δ*Pf1* mutant (Fig. 6). Although the transcriptome data indicated enhanced substrate breakdown and energy provision in the Δ*Pf1* mutant, inconsistencies were observed in the proteomes, such as GapA, AcnA, CoxB, and CcsB, which were downregulated rather than upregulated (Fig. 6). Therefore, the overall regulation of glycolysis, the TCA cycle, and respiration needs further experimental verification.

Opposing the above central catabolic pathways, genes responsible for anabolic pathways were mostly downregulated. For example, proteins GlgB and GlgC in glycogen synthesis were downregulated in the proteomes (Fig. 6). Moreover, pathways consuming intermediate products from glycolysis were mostly downregulated, including DNA synthesis (*rpiB*), tryptophan synthesis (*trpE*), and saturated fatty acid biosynthesis (AccD, FabG, *fabH*, and FabZ) (Fig. 5). Genes involved in ethanol degradation (*frmA* and *yahK* encoding alcohol dehydrogenase) were upregulated, but aldehyde dehydrogenase AldB was downregulated (Fig. 5).

**(ii) Nutrient acquisition, amino acid metabolism, and protein synthesis.** Genes responsible for nitrate transportation (*narK*) and assimilation (*nirDK* and *glnB*) were upregulated, and genes involved in amino acid metabolism, such as arginine *N*-succinyltransferase (*astA*, only at 32°C), succinylglutamate-semialdehyde dehydrogenase (*astD*, at both 4°C and 32°C), and proline dehydrogenase (*putA*) for glutamate synthesis, were upregulated (Fig. 5). This supports the idea that knocking out Pf1 could increase the assimilation of inorganic nitrogen into amino acids, suggesting a higher nutrient demand in the Δ*Pf1* mutant. Protein synthesis-related genes and proteins were mostly downregulated, including ribosomal proteins (e.g., *rnc* encoding RNase III), translation initiation factors (e.g., RNA polymerase sigma factor RpoS), molecular chaperones (e.g., *htpX* encoding heat shock protein), and tRNA synthesis (e.g., *pdsO*) (Data Sets S2B and S3B).

**FIG 7:**
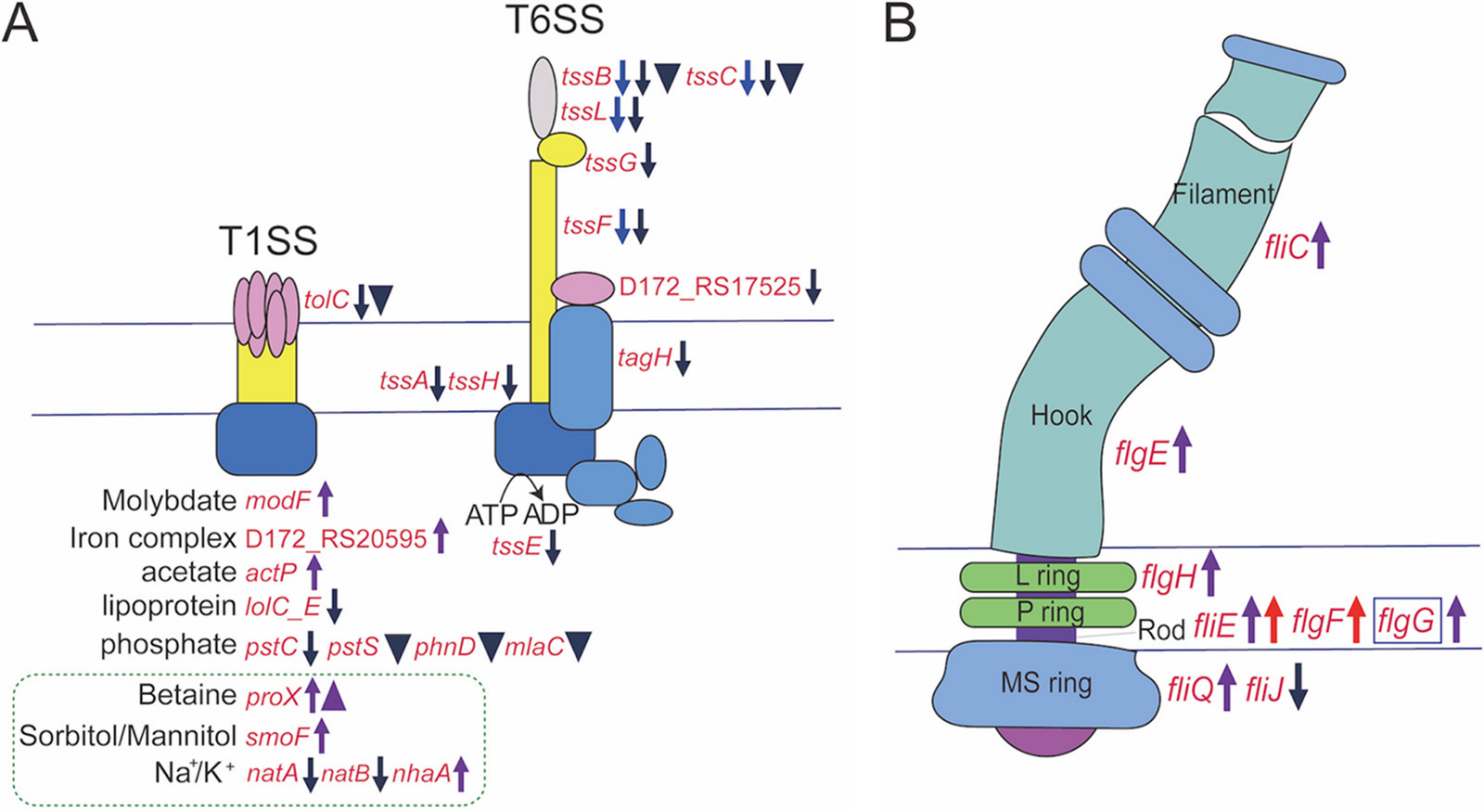
Upregulated and downregulated genes of type I/VI secretion systems and flagellar synthesis in the Δ*Pf1* mutant. (A) Type I/VI secretion systems; (B) flagellar synthesis. Genes enclosed in blue boxes represent the identified regulatory targets of CsrA in E. coli. Arrows and triangles next to the genes and proteins represent the upregulation and downregulation in the transcriptome and proteome, respectively. The colors represent different modulation outcomes: red, upregulated at 32°C; blue, downregulated at 32°C; purple, upregulated at 4°C; navy blue, downregulated at 4°C.

**(iii) Secretion systems.** Both upregulation and downregulation were detected in the type 1 secretion system (T1SS) (Fig. 7A). Upregulation included genes/proteins responsible for molybdate transport (*modF*), iron complex transport (D172_RS17525), acetate transport (*actP*), and compatible solute synthesis and transport, which involved betaine synthesis from serine (*betA* and *betB* at 4°C and 32°C, *betLT* only at 32°C), betaine transport (*proX* and ProX), sorbitol/mannitol transport (*smoF*), and 5-hydroxyectoine synthesis from aspartate (*metL* and MetE). In addition to the regulation of compatible solute biosynthesis and transport, sodium transport system ATP-binding protein (*natA*) and sodium transport system permease protein (*natB*) involved in sodium transport were downregulated, while sodium-hydrogen antiporter (*nhaA*) was upregulated (T1SS) (Fig. 5 and 7A). The above-mentioned changes indicated an influence of Pf1 on osmotic homeostasis. Downregulated genes/proteins involved in secretion included those responsible for lipoprotein-releasing ABC transporter permease (*lolC_E*) and phosphate transport (*pstC*, MlaC, PhnD, and PstS) (Fig. 7A, T1SS). In agreement with the downregulation of the phosphate transporters, the phosphate assimilation pathway was downregulated by downregulating genes encoding alkaline phosphatase (*phoA*), response regulator transcription factor (*phoP*), phosphate regulon sensor histidine kinase (*phoR*), and alkaline phosphatase family protein PhoD.

Genes responsible for the biosynthesis of the type VI secretion system (T6SS) machinery proteins were mostly downregulated, including those encoding the sheath and cell puncturing device (*tssB*, *tssC*, and *tssL* at 4°C and 32°C), the inner tube (*tssF* at 4°C and 32°C), the baseplate complex (*tssA* and *tssH*), accessory proteins (*tagH* and D172_RS17525), and the ATPase (*tssE*) (Fig. 7A, T6SS). Consistent with the transcriptome data, TssB and TssC were also downregulated in the proteomes (Fig. 7A, T6SS). The T6SS is usually responsible for injecting effector proteins (toxins) into other cells and hence is critical for pathogeny and mediating interactions between diverse taxa (40, 41). It suggests that Pf1 sRNA could activate the T6SS to enhance competition or manipulate interactions with other species to influence microbial communities.

**(iv) Motility and chemotaxis.** Genes responsible for the synthesis of the flagellar filament (*fliC*), the hook (*flgE*), the distal rod (*fliE* and *flgG*), the L ring (*flgH*), and the MS ring (*fliQ*) were upregulated at 4°C (Fig. 7B). Two genes in the synthesis of the distal rod (*flgF* and *fliE*) were also upregulated at 32°C (Fig. 7B). However, *fliJ*, involved in MS ring synthesis, was downregulated at 4°C. In addition, FlhF, a signal recognition particle (SRP)-type GTPase essential for complete flagellar synthesis (42) (Fig. 5), was downregulated at 4°C. This suggests that motility provided by the flagellar filament was influenced by the lack of Pf1 at both temperatures. However, the final effect on motility should be validated by experiments.

Genes involved in chemotaxis, including *mcp* encoding the transmembrane chemoreceptor, *cheW* encoding the adaptor, and *cheY* encoding the response regulator, were upregulated. However, Mcp, CheW, and CheY, as well as three other proteins involved in chemotaxis, i.e., kinase CheA, methyltransferase CheB, and CheR, were all downregulated in the proteomic data (Fig. 5).

**(v) Responses to stresses.** The lack of Pf1 altered the expression of genes related to environmental stimuli. The universal stress protein-encoding gene *uspE* was upregulated. In addition to universal stress responses, genes related to other specific stresses were differentially expressed in the absence of Pf1 (Fig. 5), as shown below. The protein KatE, conferring resistance to H_2_O_2_, was downregulated. EgtD, involved in ergothioneine (an antioxidant and cytoprotectant) synthesis, was downregulated. The gene *sppA*, encoding a signal peptide peptidase responsible for bacterial adaptation to hypoxia, was downregulated. Protein superoxide dismutase SOD2, involved in reactive oxygen species (ROS) elimination, was upregulated. A metallo-β-lactamase (MBL) fold metallohydrolase (Bla2), enabling resistance to β-lactam antibiotics, was downregulated. The gene *katG*, responsible for oxidizing kanamycin, was upregulated in the transcriptomes but downregulated in the proteomes. The genes *groE*, *groS*, and *ompA*, coding for heat shock proteins, were upregulated at 32°C. However, *cspA* and *cspD*, coding for cold shock proteins, were downregulated and upregulated at 4°C, respectively.

### Phenotypic validation of Pf1 sRNA influence.

**FIG 8:**
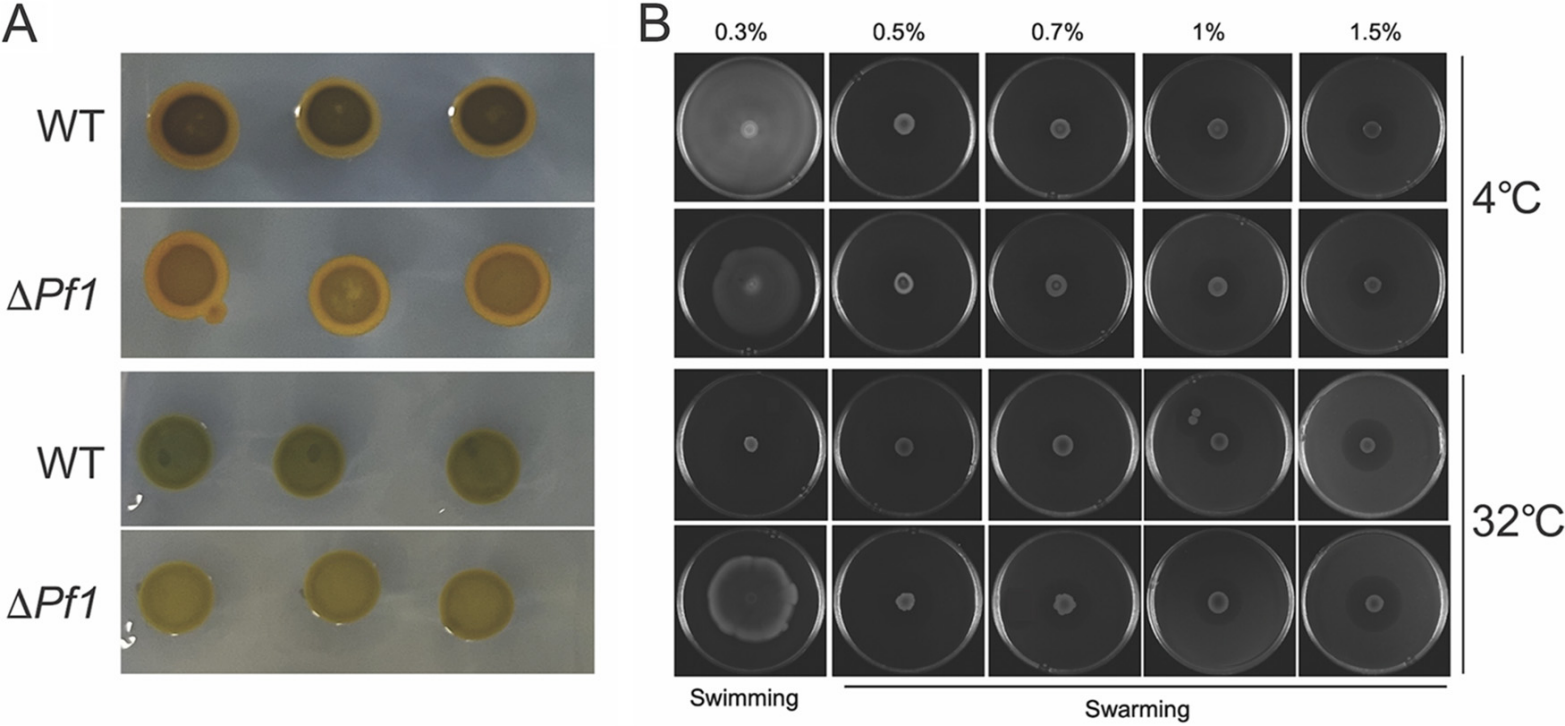
Glycogen synthesis (A) and motility (swimming and swarming) testing (B) of the WT and Δ*Pf1* mutant at 4°C and 32°C.

**FIG 9:**
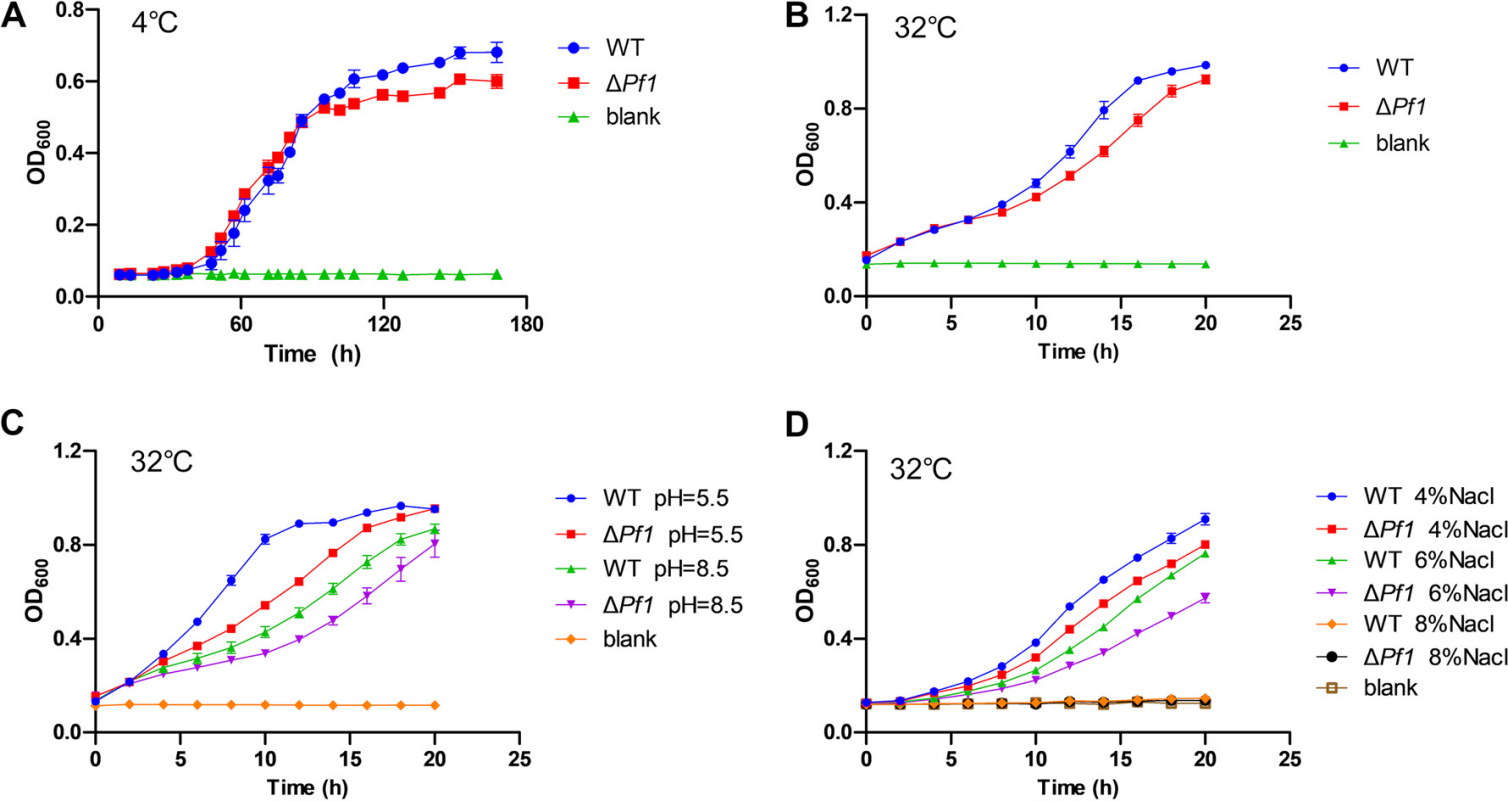
Growth curves of the WT and Δ*Pf1* mutant under different conditions, with 2216E medium as the control blank. (A) 2216E medium, 4°C; (B) 2216E medium, 32°C; (C) 2216E medium, initial pH of 5.5 or 8.5; (D) 2216E medium containing final concentrations of 4, 6, and 8% NaCl.

As revealed by the transcriptomic and proteomic data, Pf1 sRNA influenced gene expression in various aspects such as carbohydrate metabolism, motility, and stress resistance. Experiments were performed to confirm the phenotypical changes caused by Pf1. Glycogen synthesis was confirmed to be downregulated at both 4°C and 32°C (Fig. 8A; the lighter color of the Δ*Pf1* mutant developed after iodine staining), supporting the downregulation of proteins responsible for glycogen synthesis detected in the proteomes. Motility was quite different at 4°C and 32°C. Both the WT and the Δ*Pf1* mutant showed no obvious movement in plates with 0.5% to 1.5% agar, suggesting that swarming was not detected (Fig. 8B). Nevertheless, both strains were able to swim in 0.3% agar at 4°C, with the WT showing improved swimming ability compared to that of the Δ*Pf1* mutant. This phenotype agreed with the observation of the downregulation of *fliJ* at 4°C, although some other genes were upregulated at the same time. In contrast, swimming ability was completely lost in the WT but retained in the Δ*Pf1* mutant at 32°C (Fig. 8B), consistent with the transcriptomic data. Growth was impacted in the Δ*Pf1* mutant under the test conditions, including temperature (4°C, 32°C), pH (5.5 and 8.5 at 32°C), and NaCl concentration (4, 6, and 8% at 32°C), showing slower growth or less biomass compared to that of the WT (Fig. 9).

### Potential specific targets of Csr system in BSW20308.

To explore specific targets of the Csr system in strain BSW20308 compared with that in E. coli, we compared all 758 DEGs/DEPs (Data Set S6A) detected in our research to targets previously discovered in E. coli (9, 43–45). A total of 130 proteins (Data Set S6B) involved in most of the above-described processes in our research matched those Csr targets in E. coli according to the threshold used in the method. The remaining 628 unmatched genes (Data Set S6C) contained potential targets specific to *P. fuliginea* compared with E. coli, suggesting a high degree of genus/species specificity. The unmatched genes were grouped mostly into signal transduction, carbohydrate metabolism, membrane transport, cell motility, and amino acid metabolism categories (Fig. S5).

## DISCUSSION

In this study, we identified Pf1 sRNA and investigated the first Csr sRNA in *Pseudoalteromonas*. The newly discovered Pf1 sRNA appears to be conserved and widely present in most species of *Pseudoalteromonas*. However, it does not mean that species without Pf1 homologous sequences have no Csr sRNAs. The Pf1-absent species could contain Csr sRNAs with no significant sequence identity, since sRNAs evolved quickly (46). For the origins of sRNAs, *de novo* emergence, duplication, and horizontal gene transfer have been proposed (46). Since no significant sequence identity was found outside *Pseudoalteromonas*, it is possible that Pf1-like Csr sRNAs evolved *de novo*, a phenomenon reported in Saccharomyces cerevisiae (47). However, we cannot exclude the possibility that sRNAs within this genus share an ancestor with other CsrB, but the sequences are so different that this relationship is obscured. In general, sRNAs only need to be partially complementary to the target mRNA or protein, which reduces selection pressure on sequence conservation. The Pf1 sequence can successfully regulate glycogen synthesis in E. coli, suggesting that Csr sRNAs can function in different species. This may be because Pf1 contains around 21 CsrB-type conserved GGA motifs as CsrA binding sites and has a similar secondary structure (Fig. 2). This possibility agrees with previous studies in other species that Csr sRNAs are more conserved in secondary structure than in sequence (48).

Pf1 sRNA, like earlier Csr sRNAs discovered in E. coli and pathogens (49, 50), has a worldwide regulatory role, according to comparative transcriptomics/proteomics and experimental verification. Therefore, this finding further supports the idea that Csr systems are key global regulatory players not only in pathogens but also in nonpathogenic bacteria from extreme environments. Although the direct binding targets of CsrA have yet to be validated, the findings in this work reflect the end outcomes of posttranscriptional regulation caused by both direct and indirect binding. Among the differentially expressed genes and proteins at both temperatures, genes involved in central carbon metabolism, amino acid metabolism, motility and chemotaxis, compatible substance synthesis and transport, and T6SS have been proposed as regulatory targets of CsrA in BSW20308. Knocking out Pf1 did not significantly impede growth. We reason that the regulation of CsrA may involve multiple Csr sRNAs (51), such as those in E. coli (52), *V. cholera* (29), P. fluorescens (53), and S. enterica (54). Previous research in *V. cholera* revealed that multiple copies of Csr sRNAs were beneficial for responding to different signals and keeping the regulation system robust and precise (51, 55). More than one Csr sRNA could possibly be found in *Pseudoalteromonas* as well, which needs further investigation. The redundancy and compensation regulation of Csr sRNAs suggest that CsrA is critical for survival. Indeed, CsrA is under strict and multiple regulations, including autoregulation (49) and antagonism by Csr sRNAs or proteins (56). We tried to knock out CsrA in *Pseudoalteromonas* but failed (no growth was obtained after knocking out CsrA under the standard condition). Our data agree with previous reports that CsrA could not be completely removed, as with E. coli and V. cholerae (57, 58). Therefore, CsrA is indispensable in probably most species under standard conditions.

Although the knockout of Pf1 is not destructive, it has some impact on fitness under different conditions, with the Pf1 knockout lagging in growth compared to the WT under either standard or stress conditions. Meanwhile, the knockout of Pf1 also influences other physiology, such as motility and the biosynthesis of glycogen. The lack of Pf1 reduces motility at 4°C while enhancing motility at 32°C, consistent with the gene expression changes involved in flagellar biosynthesis (Fig. 7B). This suggests that Pf1 responds to temperature and regulates motility differentially. Since motility is important for free-living bacteria to migrate to appropriate microniches, this suggests that Pf1 helps bacteria survive better in cold polar oceans by maintaining motility. Transcriptomic and proteomic data also show altered viability of the Δ*Pf1* mutant under other conditions, such as the downregulation of EgtD. EgtD participates in the biosynthesis pathway of ergothioneine (ERG), which protects bacteria from redox stresses and antibiotics (59). ERG deficiency causes respiratory changes and bioenergetic inadequacies, both of which have a deleterious impact on pathogenicity (59). These findings that Pf1 regulation increases the chances of bacteria being able to survive in rapidly fluctuating and potentially adverse conditions.

### Conclusions.

We discovered and identified the first Csr sRNA of *Pseudoalteromonas*. We found that multiple physiological and biochemical processes of BSW20308 were affected after Pf1 knockout (Fig. 5; see Fig. S6 in the supplemental material). Our study laid the foundation for a better understanding of the Csr system in *Pseudoalteromonas* and revealed the importance of posttranscriptional regulation of bacteria in adaptation and response to environmental changes in the polar regions. Future research, combining experimental and computational approaches, is likely to find more examples of sRNA as a component of key bacterial regulatory pathways (60, 61).

## MATERIALS AND METHODS

### Sequence prediction and phylogeny.

The type of Pf1 was predicted by the software InvenireSRNA (62). The promoter prediction was performed using the BDGP (Berkeley Drosophila Genome Project) Neutral Network Promoter Prediction program (63). The terminator was analyzed using ARNold software (64). The secondary structures of Pf1 and CsrB from E. coli were created with mfold (65) using default folding parameters. The phylogenetic tree was constructed using the neighbor-joining algorithm in MEGA X (66).

### Promoter verification and glycogen synthesis regulation.

Predicted promoter sequences were cloned into the SpeI-digested pRU1701 plasmid in front of the promoter-less GFP by a ClonExpress II one-step cloning kit (Vazyme Co., Ltd., China), and transformed into the E. coli DE3 strain. Cells were cultured in LB with 10 μg/mL gentamicin in 24-well plates, and GFP fluorescence was detected with a microplate reader (excitation, 485 ± 20 nm; emission, 538 ± 20 nm). Cell optical density at 600 nm (OD_600_) was measured and corrected using a spectrophotometer. To verify the binding of Pf1 sRNA to CsrA, Pf1 was cloned into pET28a and transformed into the E. coli DE3 strain. Meanwhile, CsrB and CsrA from E. coli were cloned into BamHI/HindIII-digested pET28a and transformed into the E. coli DE3 strain for comparison. Escherichia coli DE3 containing the empty pET28a plasmid was used as the background control. Strains containing pET28, pET28a-CsrA, pET28a-CsrB, and pET28a-Pf1 were inoculated on a Kornberg plate (1.1% K_2_HPO_4_, 0.85% KH_2_PO_4,_ and 0.6% yeast extract containing 1% glucose) with 1 mM IPTG (isopropyl-β-d-thiogalactopyranoside) and 30 μg/mL kanamycin. The plate was incubated at 37°C overnight and then stained with iodine until a noticeable color change appeared. All experiments were performed in triplicate.

### In-frame deletion of the Pf1 gene in BSW20308.

The Pf1 gene was knocked out through homologous recombination. A plasmid for knocking out the Pf1 gene was constructed by inserting 1,000 bp of upstream and downstream sequences each into plasmid pK18-mobsacB-Ery (obtained from Xiulan Chen at Shandong University). Conjugation was performed as previously described (67). Briefly, the plasmid was transformed into E. coli strain WM3064 and cocultured with BSW20308 on a mating plate containing diaminopimelic acid (DAP). The plate was incubated overnight at 25°C. Cells were washed from the plate and resuspended in 200 μL of 2216E medium and spread onto 2216E plates with 25 μg/mL erythromycin. Colonies were picked for PCR verification of the in-frame deletion of the Pf1 gene and confirmed further through DNA sequencing.

### Transcriptome/proteome and data analysis.

To perform transcriptomic and proteomic analysis, triplicates of BSW20308 and the Δ*Pf1* mutant were grown to stationary phase at 4°C and 32°C. RNA was extracted using Invitrogen TRIzol and reverse transcribed using the PrimeScript RT reagent kit with gDNA eraser (perfect real time) in accordance with the manufacturer’s instructions. The samples were frozen at −80°C and then sequenced by Majorbio Co., Ltd., China. Genes with a log_2_ fold change of greater than or equal to 1 or less than or equal to −1 and a *P* value (adjusted) of ≤0.05 were considered significantly differentially expressed. Proteins were extracted using acetone precipitation and quantified using a data-independent acquisition (DIA) approach at BGI Genomics Co., Ltd., China. Proteins with a log_2_ fold change of greater than or equal to 1 or less than or equal to −1 and a *P* value (adjusted) of ≤0.05 were considered significantly differentially expressed. Functional annotation was performed by comparison against the KEGG, COG, GO, and NCBI nonredundant databases. CsrA targets (CDS only) identified in E. coli were downloaded from previous studies (9, 43–45) and compared with the DEGs and DEPs (CDS only) obtained in this study (see Data Set S6A in the supplemental material). BLASTP was performed to find homologous genes sharing over 40% identity and an E value below 1E–10 between the gene sets.

### Physiological experiments.

BSW20308 and the Δ*Pf1* mutant were grown overnight in 2216E medium at 25°C and 150 rpm for seed preparation. Then, suitable amounts of seed culture were inoculated in 2216E medium under different conditions to a final density (OD_600_) of 0.01. Ten repetitions of 200 μL of broth were added to a 100-well plate (model P-97). Cell density (OD_600_) was measured every 0.5 or 2 h until the death phase. Motility was verified using disk diffusion experiments with three replicates as described previously (68, 69). In brief, 5 μL of overnight culture was dropped on 2216E plates with different agarose concentrations (0.3, 0.5, 0.7, 1, and 1.5%) and then incubated at 4°C and 32°C to detect movement.

### Data availability.

The raw sequence data of transcriptome sequencing (RNA-seq) used in this study have been deposited in the Sequence Read Archive (SRA) of the NCBI database under BioProject no. PRJNA881794. The mass spectrometry proteomics data have been deposited in the ProteomeXchange Consortium via the PRIDE partner repository under data set identifier PXD036967.
