# Supplementary material for: Identification and Regulatory Roles of a New Csr Small RNA from Arctic Pseudoalteromonas fuliginea BSW20308 in Temperature Responses
Source: Microbiol Spectr. 2023 Jan 10;11(1):e04094-22. doi: 10.1128/spectrum.04094-22 (PMC9927453; doi:10.1128/spectrum.04094-22)
Supplement: Supplemental file 1 — Fig. S1 to S6. Download spectrum.04094-22-s0001.pdf, PDF file, 1.6 MB [file spectrum.04094-22-s0001.pdf]

Supplementary Information for

Identification and regulatory roles of a new Csr sRNA from Arctic *Pseudoalteromonas fuliginea* BSW20308 in temperature responses

Running title: A new Csr sRNA from *Pseudoalteromonas*

Jiao Wen[1], Li Liao*[,1,2,3], Zedong Duan[1], Shiyuan Su[1], Jin Zhang[1], Bo Chen[1]

[1]Key Laboratory for Polar Science, Ministry of Natural Resources, Polar Research Institute of China, Shanghai 200136, China

[2]School of Oceanography, Shanghai Jiao Tong University, Shanghai, China

[3]Southern Laboratory of Ocean Science and Engineering (Guangdong, Zhuhai), Zhuhai, 519000, China

*Corresponding author: Li Liao, liaoli@pric.org.cn

This supplementary file includes:

Suppl. Figs. S1 to S6

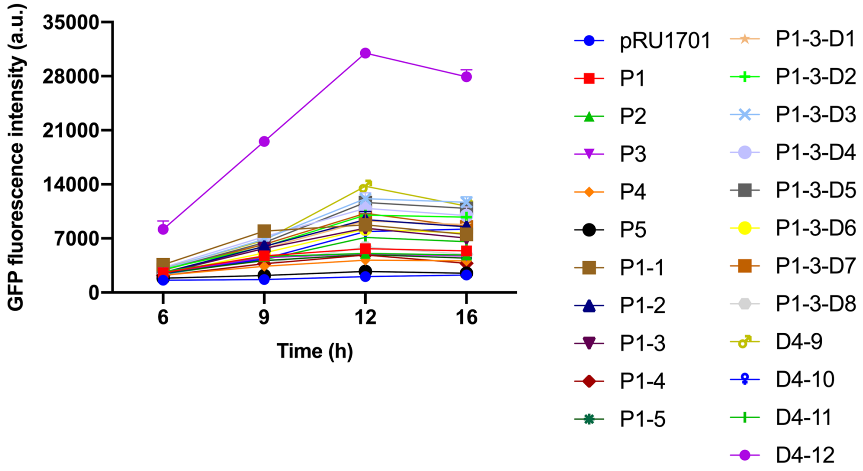

Figure S1. Predicted promoter activity validation and minimum promoter sequence identification through GFP fluorescence measurement. The promoter sequences tested are listed in Supplementary Data 1.

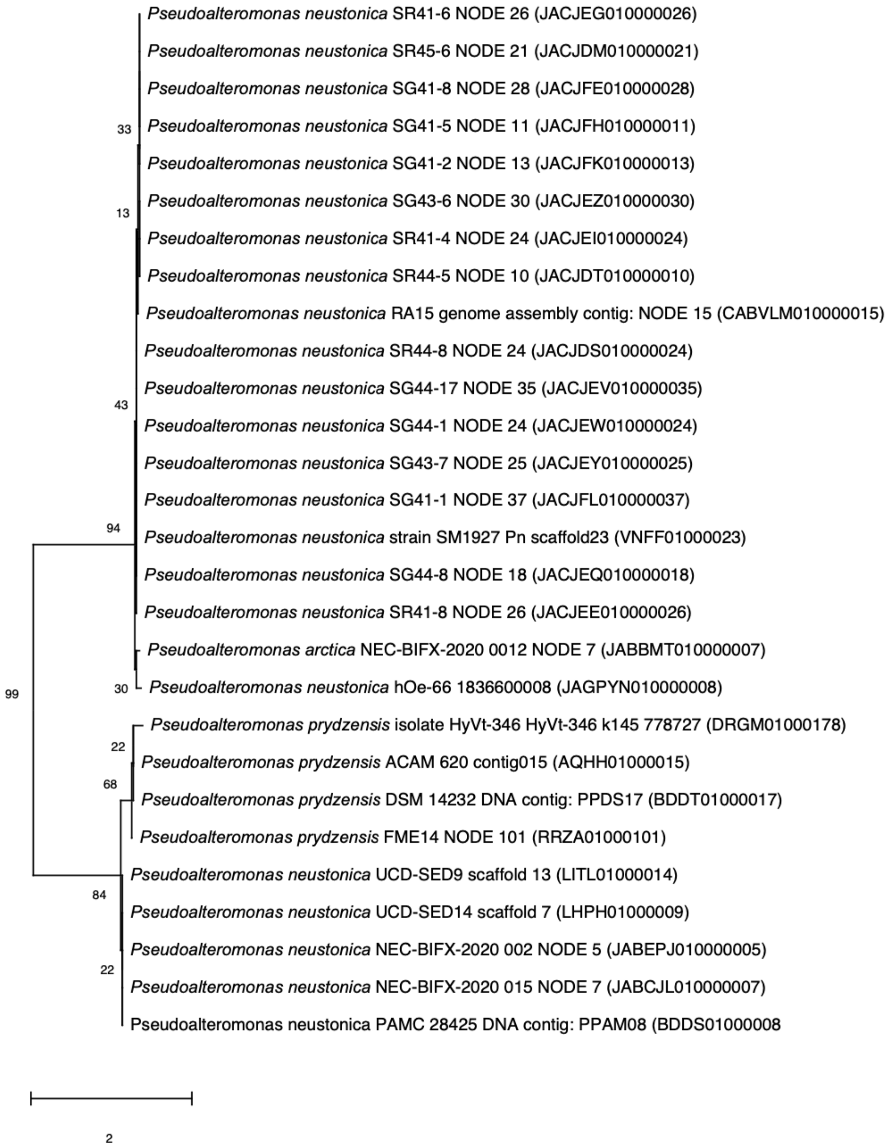

Figure S2. The phylogenetic analysis of Pf1-homologous sequences in selected species. The evolutionary relationships between species were evaluated by constructing a neighbor-joining phylogenetic tree based on Pf1-like sequences using MEGA X.

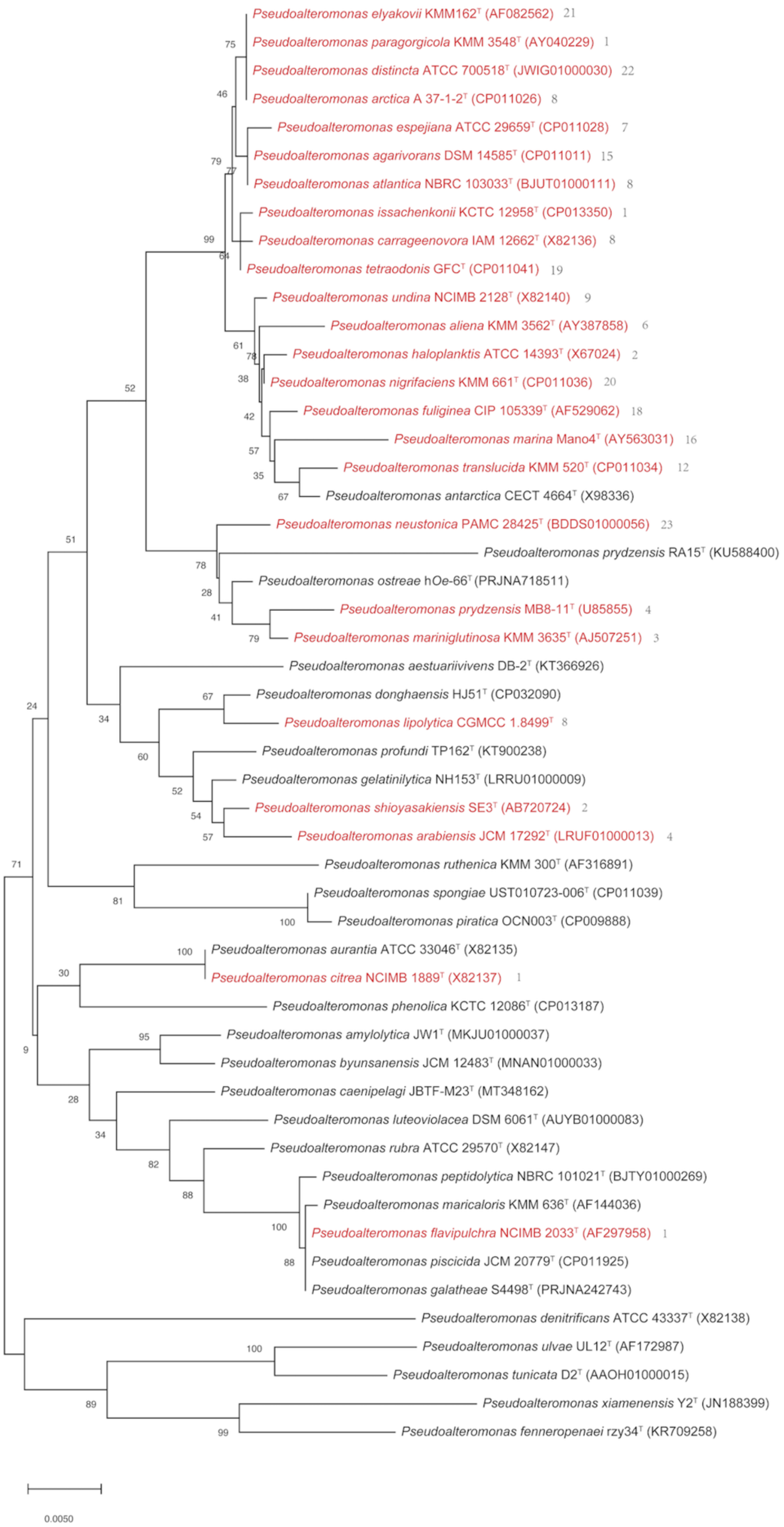
Pseudoalteromonas elyakovii KMM162T (AF082562) 21
Pseudoalteromonas paragorgicola KMM 3548T (AY040229) 1
Pseudoalteromonas distincta ATCC 700518T (JWIG01000030) 22
Pseudoalteromonas arctica A 37-1-2T (CP011026) 8
Pseudoalteromonas espejiana ATCC 29659T (CP011028) 7
Pseudoalteromonas agarivorans DSM 14585T (CP011011) 15
Pseudoalteromonas atlantica NBRC 103033T (BJUT01000111) 8
Pseudoalteromonas issachenkonii KCTC 12958T (CP013350) 1
Pseudoalteromonas carrageenovora IAM 12662T (X82136) 8
Pseudoalteromonas tetraodonis GFCT (CP011041) 19
Pseudoalteromonas undina NCIMB 2128T (X82140) 9
Pseudoalteromonas aliena KMM 3562T (AY387858) 6
Pseudoalteromonas haloplanktis ATCC 14393T (X67024) 2
Pseudoalteromonas nigrifaciens KMM 661T (CP011036) 20
Pseudoalteromonas fuliginea CIP 105339T (AF529062) 18
Pseudoalteromonas marina Mano4T (AY563031) 16
Pseudoalteromonas translucida KMM 520T (CP011034) 12
Pseudoalteromonas antarctica CECT 4664T (X98336)
Pseudoalteromonas neustonica PAMC 28425T (BDDS01000056) 23
Pseudoalteromonas prydzensis RA15T (KU588400)
Pseudoalteromonas ostreae hOe-66T (PRJNA718511)
Pseudoalteromonas prydzensis MB8-11T (U85855) 4
Pseudoalteromonas mariniglutinosa KMM 3635T (AJ507251) 3
Pseudoalteromonas aestuariivivens DB-2T (KT366926)
Pseudoalteromonas donghaensis HJ51T (CP032090)
Pseudoalteromonas lipolytica CGMCC 1.8499T 8
Pseudoalteromonas profundi TP162T (KT900238)
Pseudoalteromonas gelatinilytica NH153T (LRRU01000009)
Pseudoalteromonas shioyasakiensis SE3T (AB720724) 2
Pseudoalteromonas arabiensis JCM 17292T (LRUF01000013) 4
Pseudoalteromonas ruthenica KMM 300T (AF316891)
Pseudoalteromonas spongiae UST010723-006T (CP011039)
Pseudoalteromonas piratica OCN003T (CP009888)
Pseudoalteromonas aurantia ATCC 33046T (X82135)
Pseudoalteromonas citrea NCIMB 1889T (X82137) 1
Pseudoalteromonas phenolica KCTC 12086T (CP013187)
Pseudoalteromonas amylolytica JW1T (MKJU01000037)
Pseudoalteromonas byunsanensis JCM 12483T (MNAN01000033)
Pseudoalteromonas caenipelagi JBTF-M23T (MT348162)
Pseudoalteromonas luteoviolacea DSM 6061T (AUYB01000083)
Pseudoalteromonas rubra ATCC 29570T (X82147)
Pseudoalteromonas peptidolytica NBRC 101021T (BJTY01000269)
Pseudoalteromonas maricaloris KMM 636T (AF144036)
Pseudoalteromonas flavipulchra NCIMB 2033T (AF297958) 1
Pseudoalteromonas piscicida JCM 20779T (CP011925)
Pseudoalteromonas galatheae S4498T (PRJNA242743)
Pseudoalteromonas denitrificans ATCC 43337T (X82138)
Pseudoalteromonas ulvae UL12T (AF172987)
Pseudoalteromonas tunicata D2T (AAOH01000015)
Pseudoalteromonas xiamenensis Y2T (JN188399)
Pseudoalteromonas fenneropenaei rzy34T (KR709258)
0.0050

Figure S3. Distribution and abundance of Pf1-homologous sequences in *Pseudoalternomonas.* The evolutionary relationships between species were evaluated by constructing a neighbor-joining phylogenetic tree based on 16S rRNA genes using MEGA X. Red indicates that the species contains Pf1-homologous sequences, numbers behind represent the number of strains.

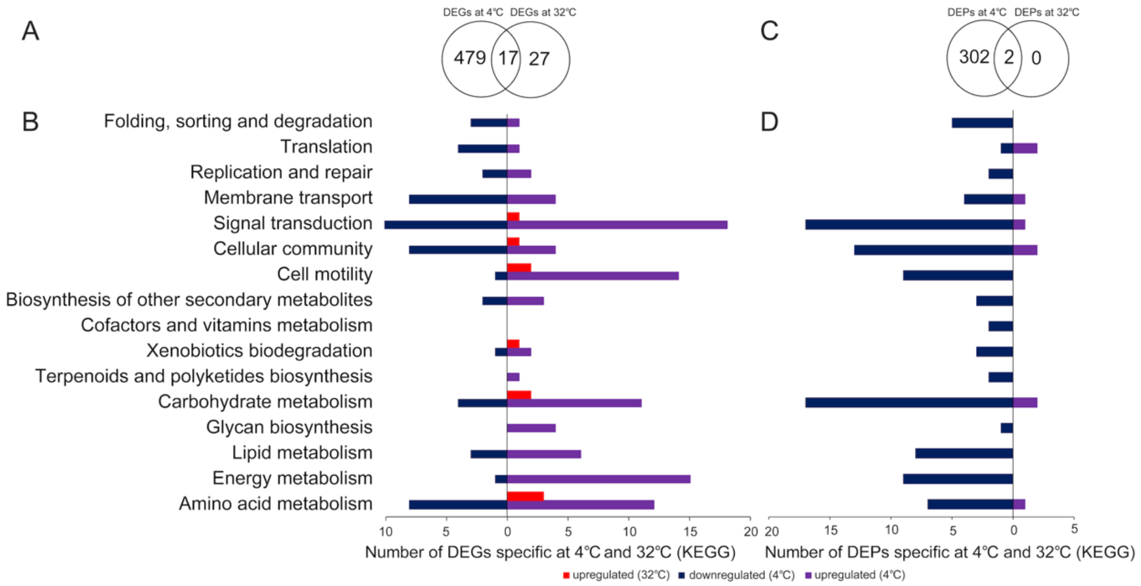

Figure S4. Comparative transcriptomics and proteomes of Δ*Pf1* normalized to the wild strain specific at 4°C and 32°C. (A) number of DEGs specific at 4°C and 32°C; (B) KEGG pathway categories of DEGs specific at 4°C and 32°C; (C) number of DEPs specific at 4°C and 32°C; (D) KEGG pathway categories of DEPs specific at 4°C and 32°C.

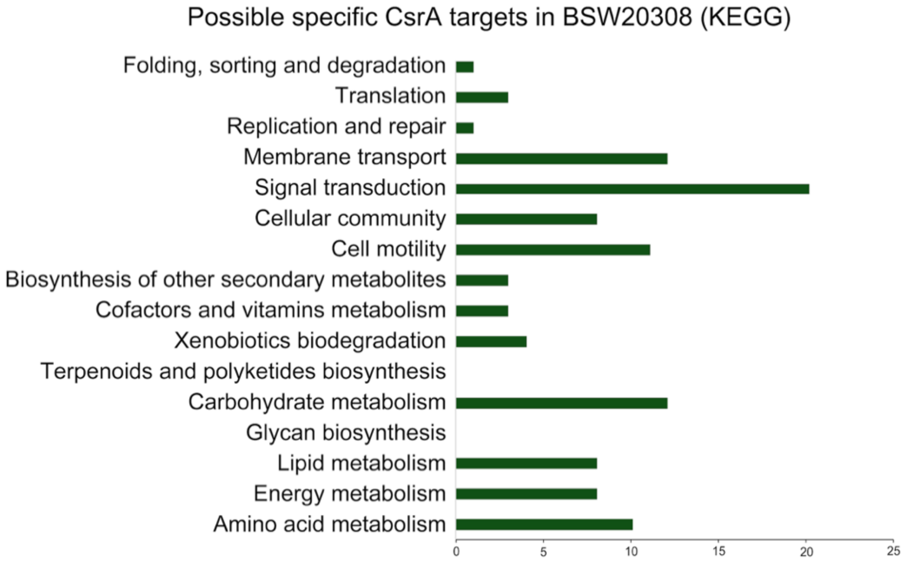

Figure S5. KEGG pathway categories of possible specific Csr targets of BSW20308.

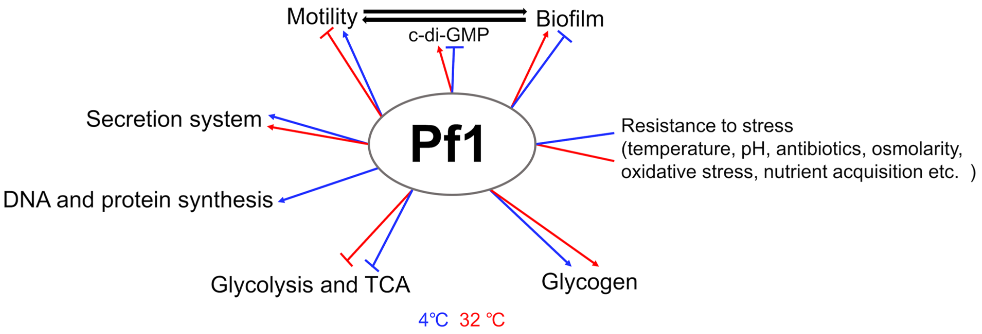

Figure S6. Illustration of major processes regulated by Pf1 in BSW20308. Arrows indicate activation of processes, while T-shaped lines represent repression. Straight lines indicate both activation and repression existing in the same process. Blue stands for 4°C and red for 32°C.
